# Emerging Biotechnology Markers of Cognitive Impairment

**DOI:** 10.1093/geroni/igab046.1719

**Published:** 2021-12-17

**Authors:** Megan Huisingh-Scheetz, Jennifer Schrack

## Abstract

The early detection of cognitive impairment is among the National Institute on Aging’s (NIA) current research priorities. Sensor-based technologies have exploded in recent years allowing remote, continuous measurement of older adults’ free-living activity. This highly granular data has stimulated exciting new research exploring how change in health can be detected remotely using novel “biotechnology” markers. Yet, this area of research is in its infancy as it relates to predicting cognitive function. This symposium will provide an overview of the sensor-cognition research landscape and will feature 5 new studies exploring the relationship between biotechnology markers and cognitive function, each with unique sensors, cognitive measures and samples. The first three presentations will report associations between accelerometry-based activity measures (chest or wrist devices) and cognitive function (assessed by diagnosis, a neurocognitive assessment, or microstructural changes on DTI) in the Baltimore Longitudinal Study on Aging, a large, NIA-funded epidemiologic dataset. The fourth presentation will report the significance of free-living hip accelerometry activity measures beyond clinically-available information in a random forest prediction model of 1-year change in Montreal Cognitive Assessment scores among urban, predominantly African-American older adults without moderate-severe dementia residing in the community. The final presentation will report associations between room-to-room transitions as detected by in-home, infrared motion sensors and mild cognitive impairment using data from a community-dwelling sample of older adults residing alone. This symposium will provide a substantial expansion of current knowledge in this research space and will be relevant to clinicians or researchers with an interest in sensor technology or dementia.

